# Special Issue “Bioactive Compounds from Natural Sources (2020, 2021)”

**DOI:** 10.3390/molecules27061929

**Published:** 2022-03-16

**Authors:** Oksana Sytar, Iryna Smetanska

**Affiliations:** 1 Institute of Plant and Environmental Sciences, Slovak Agricultural University in Nitra, 94976 Nitra, Slovakia; 2 Department of Plant Biology, Institute Biology and Medicine, Taras Shevchenko National University of Kyiv, 01033 Kyiv, Ukraine; 3 Department of Plant Food Processing, Agricultural Faculty, University of Applied Sciences Weihensteph-an-Triesdorf, Markgrafenstr 16, 91746 Weidenbach, Germany; iryna.smetanska@hswt.de

In recent decades, there has been a huge level of interest in bioactive compounds from natural sources. The large range of plant biodiversity means that there is a huge variety of bioactive compounds. Bioactive compounds are mostly specific secondary metabolites with antioxidant, inflammatory, immunomodulative potential, antimicrobial properties, etc. The bioactive compounds of classes of terpenes, flavonoids, alkaloids, coumarins, stilbenes, etc., alongside the description of some of their mechanisms of action, are important to study. It is important to note that the use of plant extracts complicates the identification of the effects of antiviral, antimicrobial or other capacities of separate biologically active compounds; additionally, the effects of activities of solute compounds in extracts can cause an additive (the synergistic effect) or antogonistic effect to come into play. In that context, the current issue is open for scientific research on the description of novel isolated bioactive compounds, as well as some of their unknown effects, which are a priority for the applied needs of humans in different areas of life.

This Special Issue includes discoveries mainly regarding the specificity of formation and effects of specific secondary metabolites of higher plants, and presents the results of the research from scientific teams based in different countries around the world in 10 original papers and three review articles.

Natural products are compounds created by living systems, and secondary metabolites are those which give species their characteristic features. These natural products include polyketides, terpenoids, phenylpropanoids, alkaloids and antibiotics [1]. Their economic significance includes their role in antimicrobial, pharmaceuticals, plant defense against herbivory, attractant, fragrance, stimulants, toxicity, plant breeding and physiological stress response. 

At the same time, the role of plant biodiversity in the variability of plant natural compounds is known well. Many natural product chemists are working to identify a large variety of novel secondary metabolites from natural materials and are eager to avoid repeatedly discovering known compounds [2].

Zhao et al., 2022 isolated natural nanoparticles of Chinese medicinal formula Naoluo Xintong—mainly composed of polysaccharides, proteins, and saponins—with typical characteristics of a two-hundred-nanometer size. It was shown that these natural nanoparticles can improve nerve function, reduce oxidative stress, and inhibit cell apoptosis in the brain. The results of the presented work can be used to develop brain protection research [3].

The work presented in this Special Issue investigates novel natural compounds as drug leads are being revitalized, particularly for tackling, antimicrobial resistance. The study of Vaičiulytė et al., 2021 evaluated the occurrence of *Thymus pulegioides* α-terpinyl acetate chemotype as a source of natural-origin α-terpinyl acetate to determine its phytotoxic and antimicrobial features. The results showed that α-terpinyl acetate was a very rare compound in the essential oil of *Thymus pulegioides*: it was found in only 35% of investigated Thymus pulegioides habitats. α-Terpinyl acetate essential oil had a highly antimicrobial effect on fungi and dermatophytes, but a lower effect on bacteria and *Candida* yeasts [4].

The large range of plant biodiversity means there is a huge variety of plant natural compounds. Content and presence differ in the various plant species depending on the climate conditions and the influence of specific abiotic and biotic factors. Norway spruce (*Picea abies* (L.) H. Karst.) is one of the most important commercial tree species distributed naturally in the Boreal and subalpine forest zone of Europe. All parts of spruce trees, including the needles, accumulate essential oils with a variety of chemical properties and ecological functions, such as modulating plant–insect communication. Kamaitytė–Bukelskienė et al., 2021 analyzed annual needle samples from 15 15–17-year-old trees (five from each of three habitats) for their essential oils and major compounds, including α-pinene, β-pinene, (1S)-(−)-α-pinene, and (1R)-(+)-α-pinene, throughout a growing season. The results showed a strong positive correlation between percentages of α- and β-pinene isomers (r = 0.69, *p* < 0.05) and between pinene isomers and essential oils: α-pinene had a stronger correlation with the essential oil (r = 0.62, *p* < 0.05) than β-pinene (r = 0.33, *p* < 0.05). Different patterns of essential oil and pinene dynamics during a growing season within separate habitats have been observed. It was suggested that such changes in essential oil composition is also connected with some genetic variables of *Picea abies* [5].

Another piece of research studies one of the several related species referred to as bearberry—*Arctostaphylos uvaursi* L. Spreng. It was revealed that the chemical characteristics and antioxidant activity of *Arctostaphylos uvaursi* L. Spreng. vary in specimens located at the southern border of the species’ geographical range in Europe. Bearberry extracts from plants growing in two different habitat types—heathlands and pine forests—showed a wide range of variation, especially in the concentration of hyperoside, corilagin, and methylartutin and the total flavonoid contents [6]. In addition to arbutin, bearberry can be a valuable source of phenolic compounds, which are mainly responsible for the antioxidant properties of extracts. The high content of phenols and high values of antioxidant parameters indicate the potential for bearberry leaves to be used as a powerful natural source of antioxidants in herbal preparations [6].

In their extensive study, Ghimire et al., 2021 investigated variations in seed characteristics, antioxidant properties, and total phenolic and flavonoid contents of sorghum collected from different ecological regions in 15 countries [7]. Using a Pearson’s correlation analysis to analyze the results revealed a wide variation range in the correlation between antioxidant activity and total phenolics content, as well as total flavonoids content, among the sorghum accessions. Variations among the accessions may provide useful information regarding phytoconstituents, antioxidant properties, and phytochemical contents of sorghum and aid in designing breeding programs to obtain sorghum with improved agronomic traits and bioactive properties [7].

Bioactive compounds in fruit and vegetables influence each other’s antioxidant activity. The absence of iron during thermal processing may influence interactions between ascorbic acid, 5-caffeoylquinic acid, and quercetin-3-rutinoside [8]. The pro-oxidant iron positively influenced the antioxidant activity in combination with the used antioxidants, while ferrous iron interacted with common in vitro assays for total antioxidant activity. The results of Engelhardt et al., 2021 indicate that the antioxidant activity of compounds is influenced by factors such as interaction with other molecules, temperature, and the minerals present [8].

Peat, also known as turf, is an accumulation of partially decayed vegetation or organic matter. It may also contain specific secondary metabolites. Therefore, using peat, which has a high natural compound content—may be be useful in agricultural applications. Black, brown, and light peat and sapropel from Lithuania were analyzed as natural sources of organic and humic substances by Jarukas et al., 2021 [9]. Significant amounts of acetohydroxamic, lactic, and glycolic acid derivatives were identified in peat and sapropel extracts. The highest amounts of fulvic acid (1%) and humic acid and humin (15.3%) were found in pure brown peat samples. This research on humic substances is useful to characterize peat of different origins to develop possible aspects of standardization, and to describe the potential of its chemical constituents.

Phytochemistry, pharmacology, and the nutraceutical profile of the Carissa species is also described in this Special Issue [10]. Carissa is a genus of shrubs or small trees native to tropical and subtropical regions of Africa, Australia and Asia. In an updated review by Dhatwalia et al., 2021, 155 research papers were analyzed. It was confirmed that *Carissa* fruits are rich in dietary fiber, lipids, proteins, carbohydrates, vitamin C, and macro- and microelements. A total of 121 compounds (35 polyphenols (flavonoids and phenolic acids), 30 lignans, 41 terpenoids, 7 steroids, 2 coumarins, and 6 cardiac glycosides) were extracted from *C. spinarum, C. carandas*, and *C. macrocarpa*. Among all chemical constituents, lupeol, carissol, naringin, carisssone, scopoletin, carissaeduloside A, D, J, carandinol, sarhamnoloside, carissanol, olivil, carinol, 3β-hydroxyolean-11-en-28,13β-oilde, ursolic acid, and carissone are the key bioactive constituents responsible for the pharmacological activities of genus *Carissa* [10].

The ethnomedicinal uses, biological activities, and triterpenoids of the *Euphorbia* species were reviewed by Kemboi et al., 2021. They present an updated and comprehensive summary of ethnomedicinal uses, phytochemistry, and the anticancer activities of triterpenoids in the *Euphorbia* species [11]. Most of the reported triterpenoids in this review belong to the tirucallane, cycloartanes, lupane, oleanane, ursane, and taraxane subclasses.

*Scrophulariae* Radix’s biological activities, together with nutraceutical and pharmaceutical applications, were reviewed by Lee et al., 2021 [12]. *Scrophulariae* Radix has an important role as a medicinal plant, the roots of which have been used to cure fever, swelling, constipation, pharyngitis, laryngitis, neuritis, sore throat, rheumatism, and arthritis for more than two thousand years in Asia. In this paper, the studies published on *Scrophularia buergeriana* and *Scrophularia ningpoensis* in the last 20 years were reviewed, and the biological activities of SB and SN were evaluated based on in vitro and in vivo studies. *Scrophularia buergeriana* had anti-inflammatory activities, immune-enhancing effects, bone disorder prevention activity, a neuroprotective effect, an anti-amnesic effect, and an anti-allergic effect; *Scrophularia ningpoensis* showed a neuroprotective effect, an anti-apoptotic effect, an anti-amnesic effect, and anti-depressant effect; and *Scrophulariae* Radix exhibited an immune-enhancing effect and cardioprotective effects via in vitro and in vivo experiments [12].

The novel method of identification for the specific secondary metabolite nathodianthrone fagopyrin is presented [13], together with a study on the role of nathodyanthrones fagopyrin and hypericin on planktonic growth and multicellular life in budding yeast [14]. The present data show that fagopyrin and hypericin uptake by the *S. cerevisiae* cells interfered with their biology both in planktonic and biofilm-like growth and accumulated in organelles and near the nucleus. A percentage of cells were unable to complete division at mitosis and initiated a new budding cycle, resulting in the formation of cell aggregates. Based on flow cytometric analysis, fagopyrin caused greater toxicity to cells compared to hypericin during a 3 h exposure. Furthermore, the autofluorescence pattern, which is found in biofilm-like structures, was severely affected in both liquid and solid media and was accompanied by abnormal morphologies. These findings suggest that the two photosensitizers have antimicrobial properties against unicellular and multicellular growth of yeast [14].

To combat the escalating levels of antibiotic resistance, novel strategies are being developed to address the ongoing demand for new natural antibiotics. The study by Wang et al., 2021 aimed to investigate amicoumacin antibiotics from the desert-derived *Bacillus subtilis* PJS by using the modern MS/MS-based molecular networking approach [15]. Hetiamacin E had strong antibacterial activity on methicillin-sensitive and -resistant *Staphylococcus epidermidis* at 2–4 µg/mL, and methicillin-sensitive and -resistant *Staphylococcus aureus* at 8–16 µg/mL. Hetiamacin F exhibited moderate antibacterial activities on *Staphylococcus* sp. at 32 µg/mL. Both compounds were inhibitors of protein biosynthesis, as demonstrated by a double-fluorescent protein reporter system [15].

This Special Issue, based on the original contributions regarding plant biodiversity and the biodiversity of plant natural compounds, will be interesting for readers of *Molecules*. Due to the high levels of interest in the current topic in the previous Special Issue, we have continued to collect unique and interesting research connected to plant natural compounds and their biological effects in this Special Issue “Bioactive Compounds from Natural Sources II.”
